# 2-(Pyrimidin-2-yl­oxy)phenol

**DOI:** 10.1107/S1600536810030448

**Published:** 2010-08-04

**Authors:** Shah Bakhtiar Nasir, Zanariah Abdullah, Zainal A. Fairuz, Seik Weng Ng, Edward R. T. Tiekink

**Affiliations:** a Department of Chemistry, University of Malaya, 50603 Kuala Lumpur, Malaysia

## Abstract

The pyrimidine and benzene rings in the title compound, C_10_H_8_N_2_O_2_, form a dihedral angle of 71.03 (7)°, with the roughly orthogonal benzene ring being folded towards one of the pyrimidine N atoms. In the crystal, pairs of O—H⋯N hydrogen bonds connect mol­ecules related by twofold symmetry into dimeric aggregates. These associate into a supra­molecular chain propagating along the *b* axis by way of C—H⋯π contacts. The chains are cross-linked by π–π inter­actions that occur between pyrimidine rings [ring centroid–centroid distances = 3.5393 (9) and 3.5697 (9) Å].

## Related literature

For background to the fluorescence properties of compounds related to the title compound, see: Kawai *et al.* (2001 ▶); Abdullah (2005 ▶). For a related structure, see: Nasir *et al.* (2010 ▶).
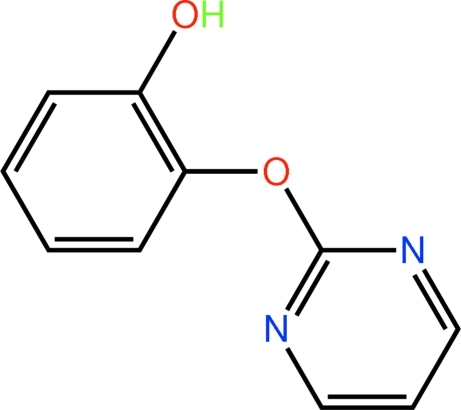


## Experimental

### 

#### Crystal data

C_10_H_8_N_2_O_2_
*M* *_r_* = 188.18
Monoclinic, 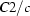

*a* = 18.0849 (18) Å
*b* = 7.3293 (8) Å
*c* = 13.3983 (14) Å
β = 92.521 (1)°
*V* = 1774.2 (3) Å^3^
*Z* = 8
Mo *K*α radiation
μ = 0.10 mm^−1^
*T* = 293 K
0.32 × 0.30 × 0.10 mm

#### Data collection

Bruker SMART APEX CCD diffractometer
Absorption correction: multi-scan (*SADABS*; Sheldrick, 1996 ▶) *T*_min_ = 0.901, *T*_max_ = 1.000
8265 measured reflections
2048 independent reflections
1569 reflections with *I* > 2σ(*I*)
*R*_int_ = 0.027

#### Refinement

*R*[*F* ^2^ > 2σ(*F* ^2^)] = 0.039
*wR*(*F* ^2^) = 0.112
*S* = 1.01
2048 reflections
130 parameters
1 restraint
H atoms treated by a mixture of independent and constrained refinement
Δρ_max_ = 0.17 e Å^−3^
Δρ_min_ = −0.18 e Å^−3^

### 

Data collection: *APEX2* (Bruker, 2009 ▶); cell refinement: *SAINT* (Bruker, 2009 ▶); data reduction: *SAINT*; program(s) used to solve structure: *SHELXS97* (Sheldrick, 2008 ▶); program(s) used to refine structure: *SHELXL97* (Sheldrick, 2008 ▶); molecular graphics: *ORTEP-3* (Farrugia, 1997 ▶) and *DIAMOND* (Brandenburg, 2006 ▶); software used to prepare material for publication: *publCIF* (Westrip, 2010 ▶).

## Supplementary Material

Crystal structure: contains datablocks global, I. DOI: 10.1107/S1600536810030448/hb5592sup1.cif

Structure factors: contains datablocks I. DOI: 10.1107/S1600536810030448/hb5592Isup2.hkl

Additional supplementary materials:  crystallographic information; 3D view; checkCIF report

## Figures and Tables

**Table 1:** Hydrogen-bond geometry (Å, °) *Cg*1 is the centroid of the C5–C10 ring.

*D*—H⋯*A*	*D*—H	H⋯*A*	*D*⋯*A*	*D*—H⋯*A*
O2—H2*o*⋯N2^i^	0.85 (2)	2.21 (1)	3.0292 (16)	163 (2)
C2—H2⋯*Cg*1^ii^	0.93	2.62	3.4424 (16)	148

Symmetry codes: (i) 
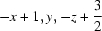
; (ii) 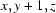.

**Fig. 1.**
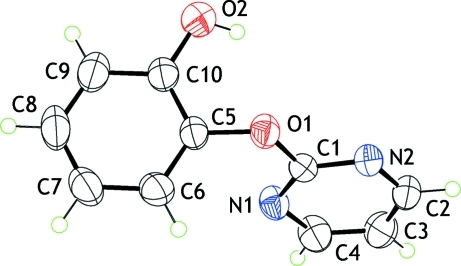
The molecular structure of (I) showing displacement ellipsoids at the 50% probability level.

**Fig. 2.**
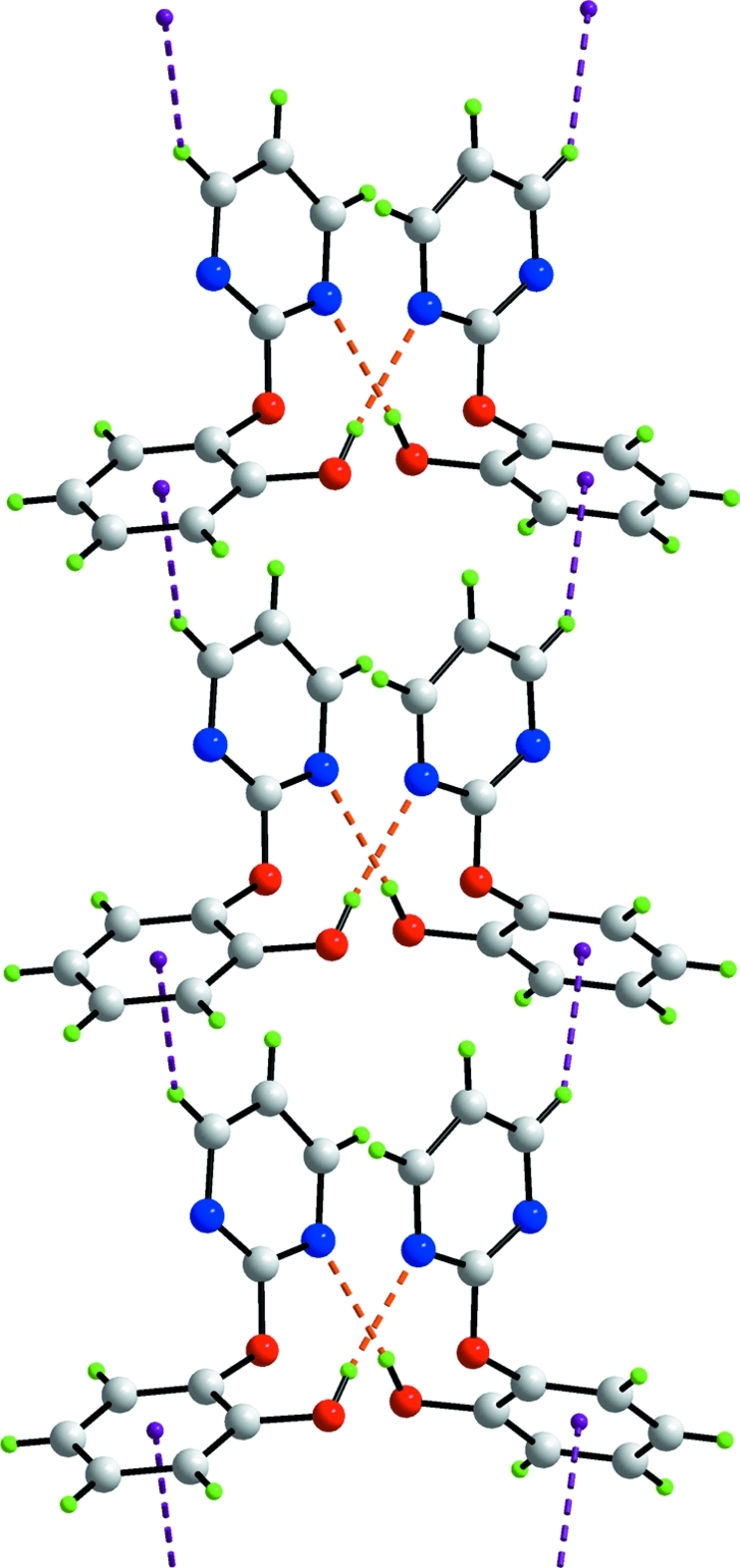
Supramolecular chain along the b axis in (I) mediated by O–H···O hydrogen bonds and C–H···π interactions, shown as orange and purple dashed lines, respectively.

**Fig. 3.**
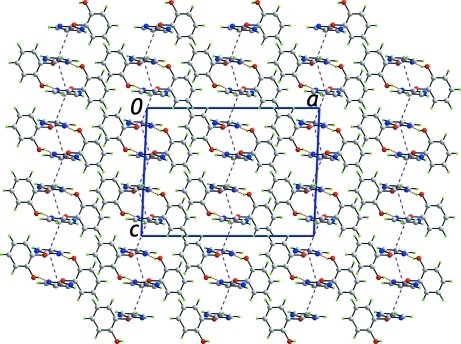
Unit-cell contents shown in projection down the b axis in (I), highlighting the connections, viaπ–π interactions, between supramolecular chains. The O–H···O hydrogen bonds and π–π interactions are shown as orange and purple dashed lines, respectively.

## supplementary crystallographic information

### Comment

Interest in the title compound relates to screening for useful fluorescence properties as seen in related compounds (Kawai *et al.* 2001; Abdullah, 2005). The molecule of (I), Fig. 1, is bent with the dihedral angle formed between the pyrimidine and benzene rings being 71.03 (7) °. The plane through the pyrimidine ring cuts through the orthogonal plane through the benzene ring, which is folded to be disposed towards the N1 atom. The overall conformation resembles that reported recently for 2-(3-methoxyphenoxy)pyrimidine (Nasir *et al.*, 2010). The hydroxy group is directed away from the pyrimidine ring, an orientation that facilitates the formation of a O–H···N hydrogen bond with a molecule related by 2-fold symmetry, Table 1. The dimeric aggregates are linked *via* C–H···π interactions occurring between a pyrimidine-H and the benzene ring. The result of these interactions is the formation of a supramolecular chain along the *b* axis, Fig. 2 and Table 1. The chains thus formed are consolidated into the crystal structure by π–π interactions occurring between the pyrimidine rings that stack along the *c* axis [ring centroid(N1,N2,C1–C4)···ring centroid(N1,N2,C1–C4)^i,ii^ = 3.5393 (9) and 3.5697 (9) Å, respectively, with inclination angles = 16 and 0 °, respectively, for *i*: 1 - *x*, *y*, 3/2 - *z* and *ii*: 3/2 + *x*, 3/2 + *y*, 1 + *z*]; Fig. 3.

### Experimental

1,2-Dihydroxybenzene (12 g, 108 mmol) was mixed with sodium hydroxide (4.32 g, 108 mmol) in several drops of water. The water was then evaporated and the resulting paste heated with 2-chloropyrimidine (2 g, 18 mmol) at 423—433 K for 5 h. The product was dissolved in water and the solution extracted with chloroform. The chloroform phase was dried over sodium sulfate; the evaporation of the solvent gave well shaped colourless blocks of (I).

### Refinement

Carbon-bound H-atoms were placed in calculated positions (C—H 0.93 Å) and were included in the refinement in the riding model approximation, with *U*_iso_(H) set to 1.2*U*_equiv_(C). The O-bound H-atom was located in a difference Fourier map, and was refined with a distance restraint of O–H 0.84±0.01 Å, and with *U*_iso_(H) set to 1.5*U*_equiv_(O).

### Crystal data

**Table:**

C_10_H_8_N_2_O_2_	*F*(000) = 784
*M**_r_* = 188.18	*D*_x_ = 1.409 Mg m^−^^3^
Monoclinic, *C*2/*c*	Mo *K*α radiation, λ = 0.71073 Å
Hall symbol: -C 2yc	Cell parameters from 2567 reflections
*a* = 18.0849 (18) Å	θ = 3.0–26.9°
*b* = 7.3293 (8) Å	µ = 0.10 mm^−^^1^
*c* = 13.3983 (14) Å	*T* = 293 K
β = 92.521 (1)°	Block, colourless
*V* = 1774.2 (3) Å^3^	0.32 × 0.30 × 0.10 mm
*Z* = 8	

### Data collection

**Table:**

Bruker SMART APEX CCD diffractometer	2048 independent reflections
Radiation source: fine-focus sealed tube	1569 reflections with *I* > 2σ(*I*)
graphite	*R*_int_ = 0.027
ω scans	θ_max_ = 27.5°, θ_min_ = 2.3°
Absorption correction: multi-scan (*SADABS*; Sheldrick, 1996)	*h* = −23→23
*T*_min_ = 0.901, *T*_max_ = 1.000	*k* = −9→9
8265 measured reflections	*l* = −17→15

### Refinement

**Table:**

Refinement on *F*^2^	Primary atom site location: structure-invariant direct methods
Least-squares matrix: full	Secondary atom site location: difference Fourier map
*R*[*F*^2^ > 2σ(*F*^2^)] = 0.039	Hydrogen site location: inferred from neighbouring sites
*wR*(*F*^2^) = 0.112	H atoms treated by a mixture of independent and constrained refinement
*S* = 1.01	*w* = 1/[σ^2^(*F*_o_^2^) + (0.0586*P*)^2^ + 0.4928*P*] where *P* = (*F*_o_^2^ + 2*F*_c_^2^)/3
2048 reflections	(Δ/σ)_max_ = 0.001
130 parameters	Δρ_max_ = 0.17 e Å^−^^3^
1 restraint	Δρ_min_ = −0.18 e Å^−^^3^

### Special details

**Table:**

Geometry. All e.s.d.'s (except the e.s.d. in the dihedral angle between two l.s. planes) are estimated using the full covariance matrix. The cell e.s.d.'s are taken into account individually in the estimation of e.s.d.'s in distances, angles and torsion angles; correlations between e.s.d.'s in cell parameters are only used when they are defined by crystal symmetry. An approximate (isotropic) treatment of cell e.s.d.'s is used for estimating e.s.d.'s involving l.s. planes.
Refinement. Refinement of *F*^2^ against ALL reflections. The weighted *R*-factor *wR* and goodness of fit *S* are based on *F*^2^, conventional *R*-factors *R* are based on *F*, with *F* set to zero for negative *F*^2^. The threshold expression of *F*^2^ > σ(*F*^2^) is used only for calculating *R*-factors(gt) *etc*. and is not relevant to the choice of reflections for refinement. *R*-factors based on *F*^2^ are statistically about twice as large as those based on *F*, and *R*- factors based on ALL data will be even larger.

### Fractional atomic coordinates and isotropic or equivalent isotropic displacement parameters (Å^2^)

**Table:**

	*x*	*y*	*z*	*U*_iso_*/*U*_eq_	
O1	0.44378 (5)	0.18501 (12)	0.65018 (7)	0.0445 (3)	
O2	0.38586 (6)	0.06384 (16)	0.82550 (8)	0.0622 (3)	
H2o	0.4144 (10)	0.156 (2)	0.8229 (16)	0.093*	
N1	0.39630 (6)	0.47232 (14)	0.61828 (9)	0.0431 (3)	
N2	0.52544 (6)	0.41044 (15)	0.63993 (8)	0.0423 (3)	
C1	0.45408 (7)	0.36583 (16)	0.63476 (9)	0.0356 (3)	
C2	0.41257 (8)	0.64865 (19)	0.60601 (12)	0.0523 (4)	
H2	0.3740	0.7306	0.5935	0.063*	
C3	0.48369 (9)	0.71379 (19)	0.61104 (12)	0.0542 (4)	
H3	0.4940	0.8371	0.6031	0.065*	
C4	0.53883 (8)	0.5881 (2)	0.62839 (10)	0.0491 (3)	
H4	0.5877	0.6283	0.6323	0.059*	
C5	0.37173 (7)	0.11373 (16)	0.64788 (10)	0.0396 (3)	
C6	0.33142 (8)	0.09337 (19)	0.55923 (12)	0.0537 (4)	
H6	0.3495	0.1381	0.5001	0.064*	
C7	0.26364 (9)	0.0057 (2)	0.55871 (14)	0.0651 (5)	
H7	0.2356	−0.0076	0.4993	0.078*	
C8	0.23810 (8)	−0.0614 (2)	0.64619 (15)	0.0653 (5)	
H8	0.1927	−0.1212	0.6456	0.078*	
C9	0.27880 (8)	−0.0416 (2)	0.73537 (13)	0.0575 (4)	
H9	0.2609	−0.0885	0.7941	0.069*	
C10	0.34627 (7)	0.04819 (17)	0.73708 (11)	0.0438 (3)	

### Atomic displacement parameters (Å^2^)

**Table:**

	*U*^11^	*U*^22^	*U*^33^	*U*^12^	*U*^13^	*U*^23^
O1	0.0343 (5)	0.0366 (5)	0.0623 (6)	0.0010 (4)	−0.0025 (4)	0.0077 (4)
O2	0.0621 (7)	0.0705 (7)	0.0534 (6)	−0.0158 (5)	−0.0041 (5)	0.0089 (5)
N1	0.0372 (6)	0.0360 (6)	0.0557 (7)	0.0015 (4)	−0.0036 (5)	−0.0019 (5)
N2	0.0337 (6)	0.0490 (6)	0.0441 (6)	−0.0023 (5)	0.0010 (4)	0.0043 (5)
C1	0.0352 (6)	0.0372 (6)	0.0343 (6)	−0.0008 (5)	−0.0012 (5)	0.0006 (5)
C2	0.0516 (8)	0.0359 (7)	0.0686 (10)	0.0031 (6)	−0.0070 (7)	−0.0024 (6)
C3	0.0606 (9)	0.0385 (7)	0.0634 (9)	−0.0089 (6)	0.0005 (7)	0.0007 (6)
C4	0.0423 (7)	0.0553 (8)	0.0499 (8)	−0.0131 (6)	0.0026 (6)	0.0023 (6)
C5	0.0336 (6)	0.0292 (6)	0.0554 (8)	0.0001 (5)	−0.0040 (5)	0.0032 (5)
C6	0.0587 (9)	0.0440 (8)	0.0570 (9)	−0.0057 (6)	−0.0124 (7)	0.0083 (6)
C7	0.0621 (10)	0.0485 (8)	0.0818 (12)	−0.0098 (7)	−0.0306 (9)	0.0079 (8)
C8	0.0411 (8)	0.0475 (8)	0.1058 (14)	−0.0096 (6)	−0.0125 (8)	0.0084 (9)
C9	0.0461 (8)	0.0508 (9)	0.0759 (11)	−0.0063 (6)	0.0066 (7)	0.0076 (7)
C10	0.0397 (7)	0.0364 (6)	0.0550 (8)	0.0006 (5)	−0.0014 (6)	0.0018 (6)

### Geometric parameters (Å, °)

**Table:**

O1—C1	1.3554 (15)	C4—H4	0.9300
O1—C5	1.4029 (14)	C5—C6	1.3741 (18)
O2—C10	1.3619 (17)	C5—C10	1.385 (2)
O2—H2o	0.852 (16)	C6—C7	1.384 (2)
N1—C1	1.3151 (16)	C6—H6	0.9300
N1—C2	1.3372 (17)	C7—C8	1.370 (3)
N2—C1	1.3302 (16)	C7—H7	0.9300
N2—C4	1.3347 (18)	C8—C9	1.383 (2)
C2—C3	1.371 (2)	C8—H8	0.9300
C2—H2	0.9300	C9—C10	1.3857 (19)
C3—C4	1.370 (2)	C9—H9	0.9300
C3—H3	0.9300		
			
C1—O1—C5	119.64 (9)	C6—C5—O1	121.09 (12)
C10—O2—H2o	109.1 (15)	C10—C5—O1	116.98 (11)
C1—N1—C2	114.63 (11)	C5—C6—C7	119.40 (14)
C1—N2—C4	114.48 (11)	C5—C6—H6	120.3
N1—C1—N2	128.66 (12)	C7—C6—H6	120.3
N1—C1—O1	119.50 (11)	C8—C7—C6	119.62 (15)
N2—C1—O1	111.84 (10)	C8—C7—H7	120.2
N1—C2—C3	122.80 (13)	C6—C7—H7	120.2
N1—C2—H2	118.6	C7—C8—C9	120.99 (14)
C3—C2—H2	118.6	C7—C8—H8	119.5
C4—C3—C2	116.65 (13)	C9—C8—H8	119.5
C4—C3—H3	121.7	C8—C9—C10	119.92 (15)
C2—C3—H3	121.7	C8—C9—H9	120.0
N2—C4—C3	122.77 (13)	C10—C9—H9	120.0
N2—C4—H4	118.6	O2—C10—C5	122.64 (12)
C3—C4—H4	118.6	O2—C10—C9	118.89 (13)
C6—C5—C10	121.62 (12)	C5—C10—C9	118.44 (13)
			
C2—N1—C1—N2	−0.6 (2)	C10—C5—C6—C7	0.1 (2)
C2—N1—C1—O1	178.72 (12)	O1—C5—C6—C7	173.48 (13)
C4—N2—C1—N1	1.30 (19)	C5—C6—C7—C8	−0.8 (2)
C4—N2—C1—O1	−178.02 (11)	C6—C7—C8—C9	0.5 (3)
C5—O1—C1—N1	0.31 (17)	C7—C8—C9—C10	0.4 (2)
C5—O1—C1—N2	179.70 (10)	C6—C5—C10—O2	178.77 (13)
C1—N1—C2—C3	−0.5 (2)	O1—C5—C10—O2	5.17 (18)
N1—C2—C3—C4	0.7 (2)	C6—C5—C10—C9	0.8 (2)
C1—N2—C4—C3	−1.0 (2)	O1—C5—C10—C9	−172.84 (12)
C2—C3—C4—N2	0.1 (2)	C8—C9—C10—O2	−179.10 (14)
C1—O1—C5—C6	73.64 (16)	C8—C9—C10—C5	−1.0 (2)
C1—O1—C5—C10	−112.73 (13)		

### Hydrogen-bond geometry (Å, °)

**Table:**

Cg1 is the centroid of the C5–C10 ring.

**Table:**

*D*—H···*A*	*D*—H	H···*A*	*D*···*A*	*D*—H···*A*
O2—H2o···N2^i^	0.85 (2)	2.21 (1)	3.0292 (16)	163 (2)
C2—H2···Cg1^ii^	0.93	2.62	3.4424 (16)	148

Symmetry codes: (i) −*x*+1, *y*, −*z*+3/2; (ii) *x*, *y*+1, *z*.
